# Real-World Performance of Artificial Intelligence in Diabetic Retinopathy Screening: A Systematic Review

**DOI:** 10.7759/cureus.108894

**Published:** 2026-05-15

**Authors:** Saira Ahmed

**Affiliations:** 1 Medicine and Surgery, Frimley Health Foundation Trust, London, GBR

**Keywords:** artificial intelligence, deep learning, diabetic retinopathy, ophthalmology, retinal screening

## Abstract

Diabetic retinopathy (DR) remains a leading cause of preventable blindness worldwide, and timely screening is essential for early detection and intervention. Artificial intelligence (AI), particularly deep learning, has emerged as a promising tool for automated diabetic retinopathy screening. This systematic review evaluates the diagnostic performance and real-world applicability of AI-based systems across diverse clinical settings. A systematic search of PubMed, Excerpta Medica database (Embase), and the Cochrane Library was conducted, supplemented by screening of Google Scholar, with study selection performed in accordance with PRISMA 2020 guidelines. Studies were included if they assessed AI systems for diabetic retinopathy detection using fundus-based retinal imaging and reported diagnostic accuracy outcomes. A total of 30 studies published between 2016 and 2025 were included. Across studies, AI systems demonstrated consistently high diagnostic performance, with most reporting sensitivities above 85% and specificities above 80%. Large-scale and real-world studies confirmed the feasibility of implementing AI in national and community screening programmes. Additionally, smartphone-based and handheld imaging systems demonstrated promising potential for expanding screening access in resource-limited settings. Despite these encouraging findings, variability between AI systems and study designs highlights the need for external validation and standardisation prior to widespread clinical adoption. AI has significant potential to enhance screening efficiency and accessibility, but further research is required to evaluate long-term clinical outcomes and integration into healthcare systems.

## Introduction and background

Diabetic retinopathy (DR) is a leading cause of visual impairment and blindness worldwide, particularly among working-age populations. Early detection through screening is essential to prevent disease progression and associated vision loss. However, traditional screening programmes rely heavily on trained graders and ophthalmologists, creating challenges in scalability and accessibility, particularly in resource-limited settings.

Artificial intelligence (AI), particularly deep learning, has emerged as a powerful tool for automated image analysis in ophthalmology. Most AI-based DR screening systems utilise deep learning and convolutional neural network architectures trained on large retinal image datasets. These systems analyse retinal fundus photographs to identify pathological features associated with DR and generate automated diagnostic classifications. Despite promising performance, important limitations remain, including the potential for false-negative results and reduced accuracy in cases with poor image quality or atypical retinal pathology. Failure to detect treatable disease remains a significant clinical concern and highlights the importance of continued validation and appropriate human oversight during clinical implementation.

Several landmark studies have demonstrated that AI systems can detect DR from retinal fundus images with high diagnostic accuracy, often comparable to expert human graders [1,2]. Subsequent studies have further validated these findings across different clinical environments, including community-based and outpatient screening settings [3-6].

Despite these advances, many early studies were conducted using retrospective datasets or controlled environments, which may limit generalisability to routine clinical practice. More recent research has therefore focused on evaluating AI systems in broader and more diverse populations to assess real-world applicability and integration into screening workflows.

In addition, variability between AI systems, including differences in model architecture, training datasets, and performance thresholds, represents an important consideration for clinical adoption. Understanding these differences is essential to ensure safe, reliable, and equitable implementation within DR screening programmes.

This systematic review aims to evaluate the diagnostic performance of AI-based systems for DR screening, with a particular focus on real-world applications across diverse clinical settings.

## Review

Methods 

A systematic literature search was conducted using PubMed, Excerpta Medica database (Embase), and the Cochrane Library. Additional studies were identified through manual screening of Google Scholar. The PubMed search strategy included combinations of the following keywords and Boolean operators: (“diabetic retinopathy” OR “diabetic eye disease”) AND (“artificial intelligence” OR “deep learning” OR “machine learning”) AND (“screening” OR “retinal imaging” OR “fundus photography”). Similar search strategies adapted to database-specific syntax were applied to Embase and the Cochrane Library. The search was restricted to studies published between January 2016 and March 2025.

Studies were included if they evaluated AI-based systems for DR detection using fundus photography-based retinal imaging and reported diagnostic accuracy metrics such as sensitivity, specificity, or area under the curve. Studies primarily focused on prognostic modelling, optical coherence tomography (OCT) or OCT angiography without fundus-based screening relevance, or economic evaluation without diagnostic performance assessment were excluded. Only peer-reviewed primary research studies were included. Review articles, editorials, conference abstracts, meta-analyses, and non-peer-reviewed preprints were excluded.

Titles and abstracts were screened for relevance, followed by a full-text review of eligible studies. Duplicate records were removed prior to screening. Study selection and data extraction were performed by a single reviewer using predefined inclusion and exclusion criteria to ensure consistency. The study selection process was conducted in accordance with PRISMA 2020 guidelines.

Data extracted included study characteristics (author, year, country, setting), study design, population size, AI system used, comparator, and diagnostic performance metrics.

Due to substantial heterogeneity in study design, patient populations, imaging modalities, AI systems, diagnostic thresholds, and reported outcome measures, a formal meta-analysis was not considered methodologically appropriate. Instead, a qualitative synthesis was conducted to allow comparison of study findings across diverse clinical and real-world screening settings.

The methodological quality of included studies was assessed using the Quality Assessment of Diagnostic Accuracy Studies-2 (QUADAS-2) tool. This tool evaluates four domains: patient selection, index test, reference standard, and flow and timing. Each study was assessed for risk of bias across these domains and categorised as having low or unclear risk of bias based on predefined criteria. The assessment was performed by a single reviewer.

Results 

Study Selection

**Figure 1:**
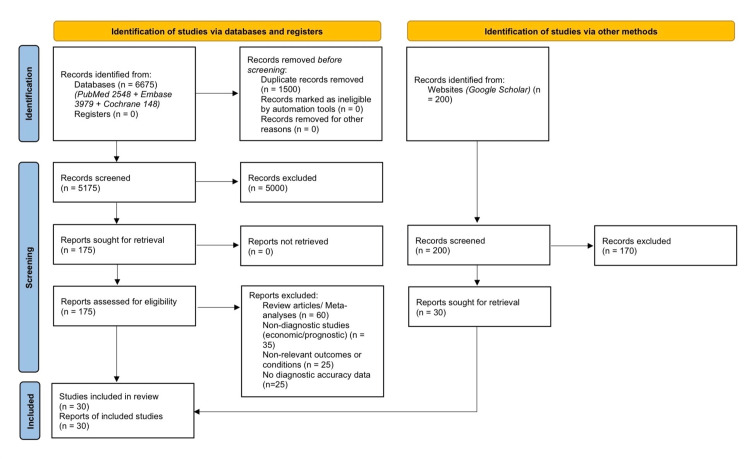
PRISMA 2020 flow diagram outlining the study selection process. Embase: Excerpta Medica database

The literature search identified 6,875 records from PubMed, Embase, the Cochrane Library, and Google Scholar. After removal of duplicates, 5,375 records were screened, with 175 full-text articles assessed for eligibility. A total of 30 studies were included in the final analysis. A PRISMA 2020 flow diagram summarising the study selection process is presented in Figure 1.

Study Characteristics

The characteristics of the included studies are summarised in Table 1, with supporting evidence drawn from all included studies [1-30].

**Table 1:** Characteristics of the included studies AI: artificial intelligence; DNN: deep neural network; AUC: area under the curve; T2DM: type 2 diabetes mellitus

Study (Year)	Country	Setting	Study Design	AI System	Comparator	Key Findings
Gulshan (2016) [1]	USA/India	Dataset	Validation	Deep learning	Ophthalmologists	High diagnostic accuracy (AUC >0.90)
Ting (2017) [2]	Multiethnic	Dataset	Validation	Deep learning	Graders	Strong performance across populations
Rajalakshmi (2018) [3]	India	Community	Diagnostic	Deep learning	Ophthalmologists	Smartphone-based screening effective
Ramachandran (2018) [4]	Australia	Clinic	Validation	DNN	Ophthalmologists	Early AI model with good accuracy
Keel (2018) [5]	Australia	Clinic	Pilot	Deep learning AI	Graders	Feasibility and acceptability demonstrated
Natarajan (2019) [6]	India	Community	Diagnostic	Offline AI	Ophthalmologists	Effective smartphone-based offline screening
Bellemo (2019) [7]	Africa	Screening	Prospective	Deep learning	Graders	High accuracy in a real-world African setting
Zhang (2020) [8]	China	Multicentre	Prospective	AI system	Ophthalmologists	Real-world validation study
Shah (2020) [9]	India	Clinic	Validation	Deep learning	Clinicians	Comparable performance to clinicians
He (2020) [10]	China	Community	Diagnostic	AI system	Ophthalmologists	Effective community screening
Wang (2020) [11]	China	Dataset	Validation	Deep learning	Graders	Automated grading system effective
Bhuiyan (2021) [12]	USA	Primary care	Diagnostic	Deep learning	Clinicians	Suitable for primary care use
Rêgo (2021) [13]	Europe	Screening	Diagnostic	Deep learning	Ophthalmologists	High diagnostic accuracy
Ming (2021) [14]	China	Community	Real-world	AI system	Clinicians	Community-based validation
Heydon (2021) [15]	UK	National	Prospective	AI system	National graders	Large-scale validation (30,000 patients)
Lee (2021) [16]	USA	Multicentre	Validation	Multiple AI systems	Experts	Variability between AI systems
Hsieh (2021) [17]	Taiwan	Clinic	Validation	VeriSee AI	Clinicians	Good clinical performance
Ipp (2021) [18]	USA	Screening	Prospective	EyeArt	Ophthalmologists	Autonomous AI detection effective
Lin (2021) [19]	China	National	Real-world	CARE AI	Clinicians	Large-scale real-world evidence
Rogers (2021) [20]	Europe	Screening	Validation	AI system	Clinicians	Handheld camera integration
Ruamviboonsuk (2022) [21]	Thailand	National	Prospective	Deep learning	Clinicians	Real-time screening in the national programme
Malerbi (2022) [22]	Brazil	Community	Diagnostic	AI system	Ophthalmologists	Smartphone-based screening effective
Pei (2022) [23]	China	Clinic	Diagnostic	AI system	Clinicians	Effective in the T2DM population
Han (2022) [24]	China	Community/Hospital	Validation	AI system	Clinicians	Works in multiple settings
Meredith (2023) [25]	UK	Population	Real-world	AI system	Screening programme	Large English population study
Vought (2023) [26]	USA	Screening	Diagnostic	EyeArt	Clinicians	Effective in screening events
Surya (2023) [27]	India	Clinic	Diagnostic	Deep learning	Clinicians	Effective for DR and glaucoma
Penha (2023) [28]	Brazil	Community	Diagnostic	AI system	Clinicians	Single-image screening effective
Wroblewski (2025) [29]	Mexico	Community	Field study	Deep learning	Clinicians	Smartphone field implementation
Brant (2025) [30]	India	National	Real-world	Deep learning	Clinicians	Strong performance in a real-world setting

The included studies were published between 2016 and 2025 and represented diverse geographic regions, including Asia, Europe, North America, and Africa. Study designs included prospective validation studies, retrospective analyses, real-world implementation studies, and pilot studies. Screening settings ranged from national DR screening programmes to community-based screening, outpatient clinics, and primary care environments. Several studies evaluated smartphone-based or handheld retinal imaging systems, while others used conventional fundus photography. Both commercial and research-based AI systems were represented, including EyeArt, VeriSee™, and deep learning models developed in academic settings [16,18].

Diagnostic Performance 

Across studies, AI systems demonstrated consistently high diagnostic accuracy. Most studies reported sensitivities exceeding 85% and specificities above 80%, with area under the curve values generally greater than 0.90 [1,2,7,21]. Several large-scale validation studies reported performance comparable to human graders, supporting the reliability of these systems in clinical screening contexts [1,2]. Despite overall strong performance, some variability in reported outcomes was observed across different study designs and populations.

Real-World and Large-Scale Studies

Several studies evaluated AI systems in real-world and large-scale screening programmes, including national initiatives. These studies demonstrated that AI can be effectively integrated into routine clinical workflows while maintaining high diagnostic accuracy across diverse healthcare settings [15,21,24,25,30]. These findings highlight the scalability and feasibility of AI-enabled screening at a population level.

Smartphone and Handheld Imaging

Multiple studies assessed AI systems using smartphone-based or handheld retinal imaging devices. These approaches demonstrated promising diagnostic performance and highlight the potential for expanding screening services in resource-limited settings, including real-world field deployment using portable imaging systems [3,6,22,28,29]. Furthermore, some systems were capable of operating offline, addressing challenges such as limited internet access and infrastructure constraints [3,6].

Comparative and Multisystem Studies

Comparative studies evaluating multiple AI systems revealed variability in diagnostic performance across different algorithms, even when applied to similar datasets [16,26,27]. These findings emphasise that AI systems are not interchangeable and require validation within specific clinical contexts. Due to heterogeneity in study populations, imaging platforms, and outcome reporting, the current evidence does not conclusively support the superiority of any single AI system for universal clinical implementation.

Quality and Heterogeneity

Considerable heterogeneity was observed across studies in terms of study design, patient populations, imaging modalities, and definitions of referable DR. Overall methodological quality was moderate to high across most studies, though variability in methodology limited direct comparison between studies. Differences in reference standards and reporting of diagnostic metrics further contributed to heterogeneity. These factors should be considered when interpreting the overall findings of this review.

Risk of Bias

The risk of bias assessment using the QUADAS-2 tool is summarised in Table 2. Overall, most studies were assessed as having a low risk of bias across the index test and reference standard domains.

**Table 2:** Risk of bias assessment using the Quality Assessment of Diagnostic Accuracy Studies-2 (QUADAS-2) tool

Study	Patient Selection	Index Test	Reference Standard	Flow & Timing
Gulshan (2016) [1]	Low	Low	Low	Low
Ting (2017) [2]	Low	Low	Low	Low
Rajalakshmi (2018) [3]	Low	Low	Low	Low
Ramachandran (2018) [4]	Unclear	Low	Low	Unclear
Keel (2018) [5]	Unclear	Low	Low	Unclear
Natarajan (2019) [6]	Low	Low	Low	Low
Bellemo (2019) [7]	Low	Low	Low	Low
Shah (2020) [8]	Unclear	Low	Low	Low
Zhang (2020) [9]	Low	Low	Low	Low
He (2020) [10]	Low	Low	Low	Low
Wang (2020) [11]	Unclear	Low	Low	Unclear
Bhuiyan (2021) [12]	Low	Low	Low	Low
Rêgo(2021) [13]	Low	Low	Low	Low
Ming (2021) [14]	Low	Low	Low	Low
Heydon (2021) [15]	Low	Low	Low	Low
Lee (2021) [16]	Low	Low	Low	Low
Hsieh (2021) [17]	Low	Low	Low	Low
Ipp (2021) [18]	Low	Low	Low	Low
Lin (2021) [19]	Low	Low	Low	Low
Rogers (2021) [20]	Low	Low	Low	Low
Ruamviboonsuk (2022) [21]	Low	Low	Low	Low
Malerbi (2022) [22]	Low	Low	Low	Low
Pei (2022) [23]	Low	Low	Low	Low
Han (2022) [24]	Low	Low	Low	Low
Meredith (2023) [25]	Low	Low	Low	Low
Vought (2023) [26]	Low	Low	Low	Low
Surya (2023) [27]	Low	Low	Low	Low
Penha (2023) [28]	Low	Low	Low	Low
Wroblewski (2025) [29]	Low	Low	Low	Low
Brant (2025) [30]	Low	Low	Low	Low

However, several studies demonstrated unclear risk of bias in the patient selection domain, particularly those based on retrospective datasets or non-random sampling methods, including studies by Ramachandran et al. [4], Keel et al. [5], Shah et al. [8], and Wang et al. [11]. These study designs may limit generalisability and introduce potential selection bias.

Similarly, unclear risk in the flow and timing domain was observed in a small number of studies, including those by Ramachandran et al. [4], Keel et al. [5], and Wang et al. [11], primarily due to insufficient reporting of participant inclusion and exclusion processes or lack of clarity regarding the timing between index testing and reference standard assessment.

Most studies were assessed as having low risk of bias in the index test domain, reflecting the use of predefined AI algorithms. The reference standard domain was also consistently low risk across studies, as most utilised expert grading or validated reference standards.

Overall, the methodological quality of included studies was considered moderate to high, with most limitations relating to study design and reporting rather than fundamental flaws in methodology.

Discussion

This systematic review demonstrates that AI systems achieve consistently high diagnostic accuracy for DR screening across a wide range of clinical and real-world settings. Landmark studies established that deep learning algorithms can perform at levels comparable to expert human graders [1,2], while more recent large-scale evaluations suggest that this performance can be maintained during routine clinical implementation [15,21]. Collectively, these findings support the growing role of AI as a clinically viable adjunct within DR screening programmes.

A major strength of the current evidence base is the increasing emphasis on real-world applicability. While many early studies relied on retrospective datasets and controlled research environments, more recent investigations have demonstrated successful deployment within national and large-scale screening programmes [15,21,25]. Studies conducted across diverse healthcare systems and populations further reinforce the potential generalisability of AI-assisted screening approaches [24-30]. Importantly, this transition from proof-of-concept research towards prospective and real-world validation represents a significant step in the maturation of AI-based screening technologies.

Despite these encouraging findings, variability between AI systems remains an important consideration for clinical implementation. Comparative studies have demonstrated differences in sensitivity and specificity across algorithms, although direct comparison between AI systems remains challenging due to variation in study design, patient populations, imaging platforms, diagnostic thresholds, and outcome reporting [16,26,27]. As a result, the current evidence does not conclusively support the superiority of any single AI system for universal clinical implementation.

From a clinical perspective, the findings of this review suggest that AI-based screening systems may be particularly valuable in healthcare settings with limited access to trained graders or ophthalmologists. Portable and smartphone-based imaging technologies demonstrated promising diagnostic performance and may help extend screening services to underserved or resource-limited populations [3,6,22,28,29]. However, the successful implementation of these systems depends not only on algorithmic performance but also on reliable image acquisition, which remains a practical challenge when using handheld or portable imaging devices. AI systems should therefore be viewed as adjunctive tools designed to support, rather than replace, clinical decision-making and specialist oversight.

The findings of this review also reflect the rapid evolution of AI performance over time. Earlier studies generally reported lower diagnostic accuracy compared with more recent prospective and large-scale evaluations, likely reflecting improvements in algorithm development, training datasets, computing power, and image standardisation [4,5,21,25]. At the same time, reliance on retrospective datasets in several early studies may partly explain the exceptionally high diagnostic performance reported in some settings and may not fully reflect the complexities encountered during routine clinical deployment [1,2].

Several limitations should be acknowledged. First, substantial heterogeneity in study design, imaging modalities, AI systems, diagnostic thresholds, and reported outcome measures limited direct comparison between studies and precluded formal meta-analysis. Second, some studies relied on retrospective datasets, introducing potential selection bias and limiting assessment of prospective clinical performance [1,2]. Third, relatively few studies evaluated long-term clinical outcomes, such as impact on vision loss, patient adherence to screening pathways, or overall screening uptake [15,21]. In addition, integration of AI into clinical practice raises important considerations beyond diagnostic accuracy alone, including workflow integration, clinician acceptance, regulatory oversight, data governance, and medico-legal responsibility.

Future research should move beyond diagnostic accuracy metrics alone and focus on long-term clinical outcomes, cost-effectiveness, workflow integration, and prospective multicentre validation across diverse healthcare settings. Further evaluation of patient acceptance and clinician trust will also be important to support safe and sustainable implementation of AI-assisted DR screening programmes.

Overall, AI has the potential to transform DR screening by improving efficiency, expanding access to care, and reducing the burden on healthcare systems. However, its successful adoption will depend not only on technological performance but also on thoughtful clinical integration, robust validation, and continued human oversight.

## Conclusions

AI systems have demonstrated consistently high diagnostic accuracy for DR screening and have rapidly evolved from proof-of-concept models to real-world clinical tools. The evidence synthesised in this review suggests that AI is not only capable of matching human graders in controlled settings but can also be effectively integrated into routine screening pathways across diverse healthcare systems. Importantly, the emergence of portable and smartphone-based imaging technologies highlights the potential of AI to extend screening beyond traditional clinical environments. This represents a significant opportunity to address longstanding disparities in access to DR screening, particularly in resource-limited and underserved populations. However, the successful implementation of portable screening platforms depends not only on the AI algorithm itself but also on the quality and reliability of image acquisition, which remains an important practical challenge when using handheld and smartphone-based devices.

However, the findings of this review also emphasise that diagnostic accuracy alone is insufficient to determine clinical value. Variability in sensitivity and specificity between AI systems and screening platforms, together with the limited assessment of long-term outcomes, underscores the need for more robust evaluation of real-world impact, including patient outcomes, cost-effectiveness, and integration into healthcare workflows. Ensuring appropriate validation and governance frameworks will be critical to maintaining patient safety and trust. Future research should focus on prospective multicentre validation studies, long-term clinical outcomes, cost-effectiveness analyses, and evaluation of how AI systems can be safely and effectively integrated into routine DR screening pathways across diverse healthcare settings. AI is poised to play a transformative role in the future of DR screening. Its successful adoption will depend not only on technological performance but also on thoughtful clinical integration, standardisation, and continued evaluation within real-world healthcare settings.
